# The discovery of dystrophin, the protein product of the Duchenne muscular dystrophy gene

**DOI:** 10.1111/febs.15466

**Published:** 2020-07-21

**Authors:** Eric P. Hoffman

**Affiliations:** ^1^ Department of Pharmaceutical Sciences School of Pharmacy and Pharmaceutical Sciences Binghamton University – State University of New York Binghamton NY USA

**Keywords:** Duchenne muscular dystrophy, dystrophin, membrane cytoskeleton, skeletal muscle

## Abstract

Duchenne muscular dystrophy was a well‐established medical and genetic enigma by the 1970s. Why was the new mutation rate so high in all world populations? Why were affected boys doing well in early childhood, but then showed relentless progression of muscle wasting? What was wrong with the muscle? The identification of the first fragments of *DMD* gene cDNA in 1986, prediction of the entire 3685 amino acid protein sequence, and production of antibodies to dystrophin, both in 1987, provided key tools to understand DMD genetics and molecular pathology. The identification of dystrophin nucleated extensive research on myofiber membrane cytoskeleton, membrane repair, muscle regeneration, and failure of regeneration. This in turn led to molecular therapeutics based on understanding of dystrophin structure and function. This historical perspective describes the events surrounding the initial identification of the dystrophin protein.

## Graphical abstract

Duchenne muscular dystrophy is the most common neuromuscular genetic disorder. This review describes the identification of the cause of the disorder in the late 1980s—dystrophin deficiency—and the emerging therapeutics enabled by increased understanding of dystrophin structure and function. Image from Duchenne, Guillaume‐Benjamin (1868). "De la paralysie musculaire pseudo‐hypertrophique, ou paralysie myo‐sclérosique". Arch. Gen. Med. Bibliothèque nationale de France. 11: 5–25, 179–209, 305–321, 421–443, 552–588.

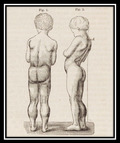

**Abbreviations**
BMD: Becker muscular dystrophy
DMD: Duchenne muscular dystrophy
NF‐κB: nuclear factor‐kappa B
RT‐PCR: reverse transcription–polymerase chain reaction
TrpE: tryptophan E gene

## *DMD* gene and identification of dystrophin

**Fig. 1:**
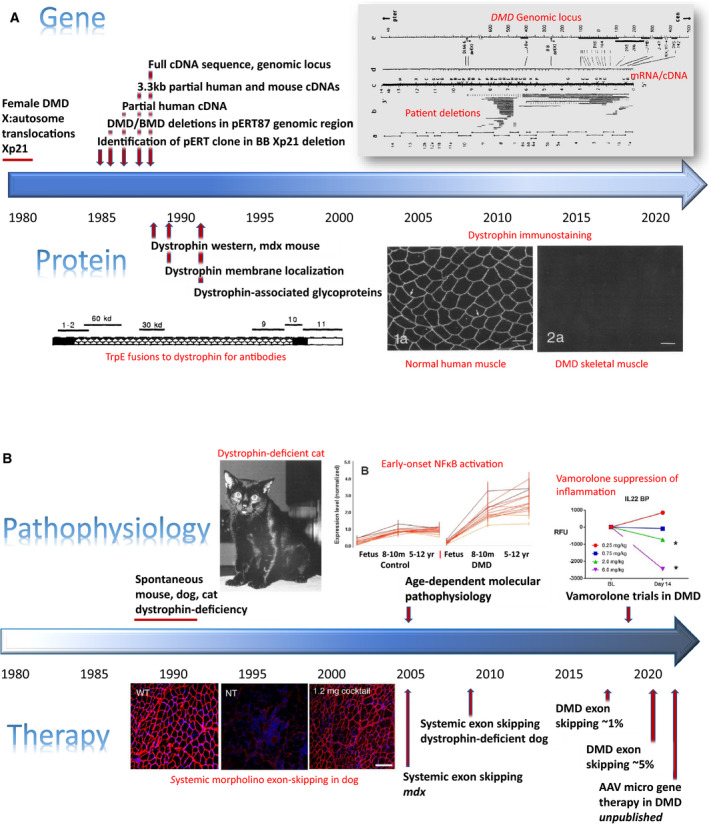
Timelines. (A) Timeline of *DMD* gene and dystrophin protein discovery. Shown is a timeline of key milestones in the identification of the *DMD* gene (top) and dystrophin protein (bottom). Citations relevant to each milestone are provided in the text. Figure inserts. DMD genomic locus (upper right) shows a schematic of the genomic locus and chromosomal walks (top), cDNA/mRNA map (middle), and patient gene deletions (bottom). Taken from Koenig* et al*. (1987) (fig. 3) [7]. TrpE fusions to dystrophin for antibodies. Affinity‐purified, region‐specific dystrophin antibodies produced against dystrophin. A schematic of the 427 kDa dystrophin protein, with its four constituent domains, is shown. Taken from Hoffman* et al*. (1990) (fig. 1) [41]. Dystrophin immunostaining. Dystrophin immunofluorescence showing membrane localization in normal skeletal muscle and loss of dystrophin in DMD muscle. Taken from Bonilla* et al*. (1989) figs 1a, 2a). Bar = 50 µm [11]. (B) Timeline of dystrophin‐enabled pathophysiology and therapeutics. Shown is a timeline of increased knowledge of the pathophysiological consequences of dystrophin deficiency and the emergence of therapeutic approaches. Citations relevant to each milestone are provided in the text. Figure inserts. Dystrophin‐deficient cat. Spontaneously occurring dystrophin deficiency in domestic cats; cats show lethal muscle hypertrophy. Taken from Gaschen* et al*. (1992) (fig. 1) [33]. Early‐onset NF‐κB inflammation. DMD muscle shows strong activation of NF‐κB ‘cell danger signal’ pathways from 8 to 10 months of age, long before obvious clinical symptoms. Taken from Chen* et al*. (2005) (fig. 1) [30]. Systemic morpholino exon skipping in dog. Rescue of dystrophin in the CXMD dog model using morpholino oligonucleotides. Taken from Yokota* et al*. (2009) (fig. 3). Bar = 100 µm [25]. Vamorolone suppression of inflammation. DMD patient sera show dose–response suppression of inflammation‐associated biomarkers. Taken from Conklin* et al*. (2019) (fig. 3) [39].

I was finishing off a PhD in *Drosophila* P‐element transformations at Johns Hopkins University in 1985 and wanting to apply recombinant DNA skills to human disease research in a post‐doc fellowship. I asked colleagues in the Johns Hopkins medical genetics group, ‘What human disease genes are close to cloning?’ They pointed me to Duchenne muscular dystrophy (DMD). Much of the groundwork had been laid for identifying the DMD gene. DMD was known to show an X‐linked recessive inheritance pattern, so the gene must be on the X chromosome (narrows down to ~ 10% of genome). In the late 1970s and early 1980s, a series of young girls with a DMD‐like clinical picture were identified, and they shared X autosome chromosomal translocations, with the X chromosome breakpoint always at the Xp21 region [1]; this suggested the DMD gene must be at Xp21 (Fig. 1A).

Lou Kunkel’s laboratory at Boston Children’s Hospital was quickly becoming a hotbed of human genetics research in DMD. Lou and his impressive Harvard MD/PhD student then, Anthony Monaco, had been working on identifying DNA in and around the putative *DMD* genomic locus at Xp21. In 1985, Lou and Tony reported isolated small DNA fragments that were deleted in a DMD patient with a cytogenetically visible deletion at Xp21 [2]. In quick succession, they reported chromosome walks (overlapping λ phage genomic clones) from one of these (pERT87) that showed DMD patient deletion breakpoints within the genomic cloned area [3, 4]. When I arrived in Lou’s laboratory in mid‐1986, with my post‐doc salary kindly provided by a Muscular Dystrophy Association Fellowship, Lou’s laboratory had just accomplished key steps toward identification of the DMD gene. By late 1986, Tony had identified potential conserved exons within the genomic walk and used one to identify the first partial cDNA (RNA) clone from human fetal skeletal muscle that detected multiple putative exons within the genomic walk [5]. This cDNA clone also suggested the full‐length RNA detected in skeletal muscle was quite large, ~ 16 kb.

There were a lot of laboratories working on DMD, and the race was on to both clone and sequence the full RNA (cDNA), decode the encoded protein, and start characterizing the protein product using antibody reagents. I made a mouse heart cDNA library and cloned the corresponding murine partial cDNAs using the human cDNA as a probe. By mid‐1987, Tony and I had extended the overlapping human and mouse cDNAs, and reported the first 3.3 kb of mRNA coding sequence of the *DMD* gene mRNA and predicted protein [6]. The predicted amino acid sequence showed extensive repeated α‐helical regions with occasional helix‐breaking residues, suggesting that the encoded protein might adopt an α‐helical bundle conformation.

Michel Koenig from Strasbourg, France, soon joined the laboratory as a post‐doc, and we worked together with Tony and Lou to clone the entire 14 kb human cDNA and published an additional 1.7 kb of sequence data for the 5’ end (amino terminus of the predicted protein), bringing total sequence coverage to ~ 5 kb (of 14 kb) in both human and mouse in mid‐1987 [7]. This paper illuminated the very large size of the *DMD* genomic locus (> 2 megabases), defining the *DMD* gene as the largest gene known—an honorific position that it retains to the current day (Fig. 1A) [7]. This paper also showed the diversity, frequency, and preferential localization of deletion mutations, including the hotspot for initiation of deletions near the center of the gene. Michel and Tony continued to work on completing the sequence of the complete human cDNA, a feat completed the following year [8]. In parallel, I began working on making antibodies to identify the protein product of the *DMD* gene.

As we had published cDNA and predicted protein sequence, many laboratories began synthesizing peptides against our published sequences with the goal of making antibodies to identify the encoded protein. While I had come out of a *Drosophila* PhD well‐versed in molecular genetic methods, I was new to antibody and protein work. I felt that I was poorly positioned to compete with other expert biochemical laboratories doing peptide and antibody work, so I thought I would instead leverage the extensive set of cDNA clones we had assembled for both mouse and human to construct and express large fusion proteins as antigens for polyclonal antibodies. In asking around the Enders Building at Boston Children's Hospital, some noted the very high levels of fusion proteins that could be generated by bacterial tryptophan E gene (TrpE) fusions [9]. I initially cloned two large segments of the mouse dystrophin cDNA encoding 60 and 30 kd fragments of the putative *DMD* locus protein product (Fig. 1A). The TrpE fusions were highly insoluble, forming precipitates in the bacteria that were then purified by a simple lysis and spin‐down of the precipitate. Between 5 and 25 mg of fusion protein were isolated from 100 mL induced bacterial culture at about 80% purity. Precipitates were solubilized in high SDS, SDS/PAGE was carried out, gel bands were visualized and excised using a simple cold KCl staining, and then, the fusion protein was electroeluted out of the band in SDS/PAGE buffer. I immunized rabbits in house, but as these were my first efforts at generating antibodies, I contacted Nigel Fleming, a post‐doc at McLean Hospital in the Harvard system, to immunize sheep as well.

As the immunized animals were building up antibodies, I looked to make affinity columns with the fusion proteins. However, the insolubility of the fusion proteins complicated coupling to Sepharose columns. I hoped that I could stay in antibody excess while carrying over some inevitable non‐column‐bound fusion protein. Indeed, this worked, where I ended up with fusion protein/antibody complexes that amplified the immunoblot signal, leading to identification of the dystrophin protein [10]. This first paper confirmed the large size of the protein predicted by cDNA cloning and sequencing (427 kDa) and confirmed the absence of dystrophin in muscle biopsies from DMD patients expected by the recessive inheritance (loss of function). The name ‘dystrophin’ was introduced in this paper (derived from ‘muscular dystrophy’), and this name was then broadly adopted. In 1988, dystrophin immunostaining showing localization at the myofiber membrane in normal skeletal muscle, and absence in DMD muscle, was published in collaboration with Eduardo Bonilla at Columbia University [11] (Fig. 1A).

The *mdx* mouse model was a potential model of muscular dystrophy that had arisen sporadically at Jackson Laboratory, but genetic mapping had placed the potential *mdx* gene locus in an area seemingly inconsistent with the human *DMD* genetic map [12]. However, a *DMD* cDNA clone used in the mouse a few months later suggested that the *mdx* mouse locus could indeed be orthologous to the *DMD* human locus [13]. In the initial dystrophin paper, skeletal muscle from *mdx* mice showed the absence of dystrophin protein, further bolstering the likelihood that *mdx* mice and *DMD* patients shared the same genetic and primary biochemical defect [10]. The specific mutation causing the original sporadic *mdx* allele was later identified (stop codon in exon 23) [14].

The Kunkel laboratory, as well as the broader DMD research community, had a strong culture of sharing of reagents and information, often before publication, and we quickly broadly distributed TrpE fusion constructs and proteins, as well as sheep antibodies. Louise Nicholsen of Newcastle University [15] and Glenn Morris of N.E. Wales Institute [16] used the TrpE fusion proteins to make a series of monoclonal antibodies and then distribute these to the scientific community via Novocastra Laboratories and later the Iowa Hybridoma Bank. Nigel Fleming, the instructor at McLean with the sheep, asked if he could start a new biotech based on use of the dystrophin antibodies for clinical testing of patient muscle biopsies. Genica Pharmaceuticals (later renamed Athena Diagnostics) was later sold in 2011 for $740 M to Quest Diagnostics through Goldman Sachs and remains one of the larger molecular diagnostics companies in the neurology space.

## DMD pathophysiology and therapy

A PubMed search for ‘dystrophin’ (June 2020) returns 8448 publications. What are some key deliverables of 32 years of dystrophin‐enabled research?

Beauty is in the eyes of the beholder. With this caveat that these are my personal assessments, I feel that the intersections of basic, translational, and clinical research around dystrophin have been particularly illuminating and impactful.

### Myofiber membrane cytoskeleton

The identification of dystrophin nucleated the study of the membrane cytoskeleton of myofibers, with the subsequent work on the dystrophin‐associated glycoprotein complex and other types of muscular dystrophies associated with components of this complex creating a fertile field of discovery [17, 18] (Fig. 1B). Indeed, nearly 1500 publications have appeared defining and citing the complex glycoprotein network associated with dystrophin, connecting the basal lamina of myofibers through the plasma membrane. This in turn has defined much of the function of dystrophin; it is clearly required for membrane stabilization, but also required for assembling the many dystrophin‐associated proteins into a large macromolecular complex that anchors the myofiber to the extracellular connective tissue, with unique glycosylation moieties specialized for this function [19].

The cellular regulation of dystrophin expression appears to be a key aspect of the plasticity of muscle (hypertrophy and atrophy), with 67 microRNA binding sites in the highly conserved 3′UTR (3.6 kb), many associated with inflammation and remodeling [20]. Skeletal muscle is one of the largest organ systems in the body, and the constituent myofibers show dramatic adaptation based on the demands placed on muscle by the body. The myofibers need strong connections to the basal lamina to carry out their function of moving the body but also need to tear down and rebuild these connections to respond to physiological demands; dystrophin seems to be a cornerstone of myofiber remodeling.

### The transition to therapy

The identification of the *DMD* gene and dystrophin protein led to hopes for new therapeutic approaches that addressed the primary defect. Intrinsic features of both the gene and protein slowed progress in translation of molecular understanding to effective therapeutics. The *DMD* gene is the largest in the human genome (2 300 000 base pairs, where a typical gene is perhaps 30 000 base pairs). It is technically challenging to harness and work with a gene that large. The dystrophin mRNA is 11 000 bases and is much too large to fit in gene therapy vectors. The dystrophin protein is also large (427 kDa) and requires a specialized intracellular structural niche within myofibers throughout the body (recalling that myofibers account for more cell volume in the body than any other cell type). The required tools to translate DMD molecular knowledge to therapeutics were in hand, but terribly cumbersome to use. Unfortunately, these technical hurdles led to a 30‐year lag between gene/protein identification (~ 1990) to first successful efforts at dystrophin‐focused therapeutics.

While the *DMD* gene, mRNA, and protein were ‘difficult tools’, two opportunities opened up that facilitated recent advances in therapies: Becker dystrophy and the ‘semifunctional’ dystrophins, and increasing knowledge of the progressive pathophysiology of DMD. For the latter, dystrophin‐deficient muscle functions for quite some time relatively well, and dystrophin‐deficient heart functions reasonably well for decades. Can the process of the failure of muscle regeneration be understood, and the process slowed or stopped?

### Becker muscular dystrophy

I think the discoveries regarding the clinically milder Becker muscular dystrophy (BMD) have been illuminating at multiple levels. From some of the earliest *DMD* gene mutational studies, it was seen that Duchenne and Becker patients showed overlapping deletion mutations and that DMD patients could in fact have much smaller deletions than Becker patients [4]. Immunoblot data of patient muscle biopsies clearly showed that DMD patients showed typical 'loss‐of‐function' consistent with recessive inheritance (loss of the dystrophin protein from muscle) whereas BMD patients showed present, but abnormal dystrophin (abnormal molecular weight and/or quantities) [21]. The deletion breakpoints were carefully characterized in three DMD and three Becker patients, and the ‘reading frame hypothesis’ developed [22]. The exons remaining in a DMD patient are spliced together into a mRNA transcript, but the exons neighboring the deletion do not share the same reading frame, leading to a frameshift, and premature truncation of translation of dystrophin. Such out‐of‐frame mRNAs are unstable due to nonsense‐mediated decay, and the low amounts of truncated dystrophin are generally nonfunctional (consistent with loss of function). Becker patients showed deletion mutations where exons neighboring the deletion shared the same reading frame, and the resulting mRNA transcript could support full translation of the dystrophin protein (and avoid nonsense‐mediated decay), although the resulting Becker dystrophin was lacking amino acids corresponding to the deleted exons. Genotype/phenotype/biochemical studies of series of Becker patients showed that large regions of the central rod domain of dystrophin could be deleted or duplicated, yet some of these patients showed mild phenotypes and others more severe phenotypes [23].

**Fig. 2:**
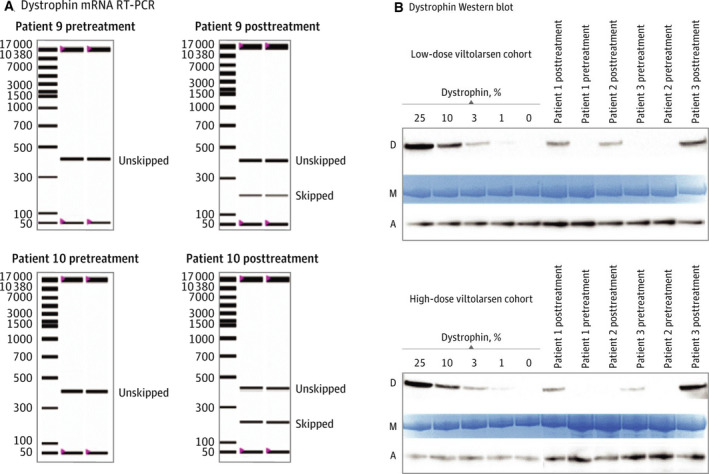
Dystrophin rescue by exon skipping in a viltolarsen clinical trial in DMD. (A) reverse transcription–polymerase chain reaction (RT‐PCR) of participant muscle biopsies taken before treatment and after treatment with viltolarsen. RT‐PCR products showing unskipped ‘out‐of‐frame’ mRNA transcript were seen pretreatment, while viltolarsen‐induced exon skipping led to a smaller skipped ‘in‐frame’ mRNA. (B) immunoblots for dystrophin [D], with protein loading controls for myosin heavy chain, M and alpha‐actinin, A. Standard curves for dystrophin are shown from mixed normal and DMD skeletal muscle samples. Clinical trial participant muscle biopsies, pretreatment and post‐viltolarsen treatment, were tested in a blinded manner. Pretreatment biopsies showed no dystrophin, whereas post‐treatment biopsies showed viltolarsen‐induced ‘Becker‐like’ dystrophin rescue. Taken from Clemens* et al*. (2020) (fig. 2) [27].

The DNA/protein/clinical correlations in BMD quickly expanded to hundreds of patients, with studies leading to a dystrophin protein ‘deletion’ map. It became clear that the dystrophin protein could sustain enormous damage to its primary structure, yet still retain some or much biochemical function, evidenced by a milder clinical course of the Becker dystrophy patient. This in turn led to therapeutic strategies to change an out‐of‐frame Duchenne gene mutation into an in‐frame gene mutation using ‘exon skipping’ (modulation of RNA splicing using morpholino oligonucleotide drugs). Successful systemic rescue of high levels of dystrophin was shown in the *mdx* mouse using morpholino chemistry in 2005 [24] and in dystrophin‐deficient dogs in 2009 [25] (Fig. 1B). Translation to human clinical trials led to rescue of about 1% normal dystrophin levels in DMD boys in 2011 [26] and about 5% in 2020 (Fig. 2) [27]. Importantly, the dystrophin protein was imparted a status by the FDA enjoyed by few proteins—surrogate biomarker outcome measure sufficient for drug approval. Evidence of some rescue of dystrophin in patient muscle by exon skipping has been defined by FDA as sufficient for accelerated regulatory approval, without the typical requirement of clear evidence of clinical benefit.

The emerging amino acid maps of domains of dystrophin and their roles in myofiber cell biology led to the creation of ‘microdystrophins’ now in multiple gene therapy clinical trials (clinicaltrials.gov NCT03362502; NCT03368742; NCT03375164), but not yet published at the time of writing. It seems that FDA may not accept microdystrophins as a surrogate biomarker outcome measure sufficient for drug approval; clinical benefit must be shown.

Therapeutic approaches with both Becker‐like (exon skipping) and microdystrophins retain some function of normal, full‐length dystrophin. However, they do not retain all function. In the context of BMD patients, the same Becker‐like dystrophin (ex. deletion of exons 45–47) leads to a variable clinical phenotype with variable amounts of dystrophin in patient muscle [20, 28]. Thus, it is expected that the clinical response to both exon skipping and gene therapy will be variable from patient to patient.

### Molecular pathophysiology of DMD as a progressive disease

Soon after the initial discovery of dystrophin, we found that dystrophin is normally relatively early in fetal life; high levels were seen by 16‐week gestation in skeletal muscle and by 12‐week gestation in fetal heart [29]. Fetal dystrophin‐deficient skeletal muscle showed no evidence of pathology by mRNA profiling, but nuclear factor‐kappa B (NF‐κB)‐related inflammatory pathways were strongly activated soon after birth [30] (Fig. 1B). These observations led to the realization that the loss of dystrophin only initiates a process in skeletal muscle that takes years to lead to weakness and disability. Understanding the molecular, cellular, and clinical underpinnings of the *progressive* nature of the disease has driven much of my own ‘post dystrophin’ research efforts. There have been many dystrophin‐enabled observations that suggested that it is the downstream consequences of dystrophin deficiency that drive the relentless muscle wasting and loss seen in all DMD boys. I worked with veterinary pathologists and neurologists to identify multiple lines of cats and dogs showing loss of dystrophin in skeletal muscle—for example, animal models of DMD [31, 32, 33, 34]. Humans, dogs, cats, and mice lacking dystrophin in skeletal muscle show similar cellular defects (membrane damage leading to cycles of degeneration/regeneration of myofibers). However, dogs and human dystrophin‐deficient muscle generally progresses to fibrofatty infiltration (muscle wasting), whereas cat and mouse muscle generally do not. That said, individual muscle groups could show strikingly different age‐related changes. For example, the sartorius muscle is very well‐preserved in both dog and human dystrophin deficiency, becoming ‘super‐normal’, while adjacent muscles show extensive fibrofatty replacement [35, 36]. Despite the markedly different ‘responses’ of individual muscles to long‐term loss of dystrophin, all muscles start out the same; dystrophin is expressed in all normal skeletal myofibers early in fetal life and is lost in all dystrophin‐deficient muscles in all organisms at this same early time point.

What are the ‘downstream’ consequences of dystrophin deficiency that take years to develop to the point of muscle weakness and wasting, and can these processes be slowed or mitigated? mRNA profiling studies of patient muscle biopsies showed initiation of intramuscular inflammation cascades soon after birth (activated tissue dendritic cells, expression of Toll‐like receptor 7, and NF‐κB), years before the onset of clinical symptoms [30]. Molecular pathways associated with tissue fibrosis were activated later in symptomatic patients [transforming growth factor (TGF)‐beta pathway, expression of TGF‐beta type II receptor and apoptosis signal‐regulating kinase 1 proteins on subsets of mature DMD myofibers]. Dystrophin‐deficient muscle seemed unable to mature correctly, with failure of the acquisition of glycolytic and oxidative metabolic capacity seen during normal human muscle development; this suggested an age‐related metabolic insufficiency. In summary, dystrophin deficiency initiates innate immunity ‘danger signals’ (likely a direct result of membrane instability and cytoplasmic leakage), with downstream activation of fibrosis pathways and metabolic insufficiency that are associated with disease progression [37]. The mechanism of action of corticosteroids (deflazacort and prednisone), currently considered standard of care in DMD, is thought to be through inhibition of NF‐κB pathways in dystrophin‐deficient muscle. Vamorolone, a partial agonist of the glucocorticoid receptor, has been shown to be a potent NF‐κB inhibitor in preclinical *mdx* and *in vitro* studies [38], has shown dose‐responsive normalization of serum pro‐inflammatory proteins in DMD boys [39], and has shown preliminary evidence of improvement of muscle strength and endurance in an open‐label clinical trial [40] (Fig. 1B). Importantly, vamorolone appears to show fewer of the severe safety concerns typically observed with chronic corticosteroid treatment.

This emerging understanding of complex age‐dependent and muscle‐dependent tissue pathology has important implications for therapeutic efforts. For all DMD experimental therapeutic approaches, the target tissue of the drug or intervention is skeletal muscle and constituent myofibers. But in DMD patients, many specific muscles show an early progression to fibrofatty replacement—the myofiber target tissue may no longer be available to the drug to exert potential benefit. With this model of variable age‐related and muscle‐specific disease progression, it is predicted that the best efficacy of any intervention may be seen in younger DMD boys (newborn to 5 years) where most muscle tissue remains better preserved. It is for this reason that clinical trials of vamorolone were done in younger DMD boys (4–< 7 years) [40], and the viltolarsen exon skipping trial was done in boys (4–< 9 years of age) [27].

This model also predicts that efficacious therapies likely need to target multiple pathways, including inflammation, mitochondrial function, and fibrosis (and failed regeneration). Indeed, current therapeutic efforts at dystrophin replacement all focus on semifunctional dystrophin (Becker‐like or microdystrophins) where inflammation and other pathways are likely to still be activated. Indeed, all dystrophin replacement clinical trials still require commensurate corticosteroid treatment to mitigate effects of inflammation (despite the severe side effects associated with these drugs).

## Summary

Looking forward, the enormous *DMD* gene and enigmatic dystrophin protein will continue to present us with challenges in our efforts to understand the biology, and aid patients via therapeutics. We understand the gene mutations, the effects on dystrophin, and many features of the biochemical role of dystrophin in muscle. Indeed, the identification of the dystrophin gene and protein heralded the era of human disease genomics that has dramatically increased our understanding of human genetic disease. However, we do not understand the downstream consequences of dystrophin deficiency in a cell and its surrounding tissue. Why are some muscles ‘spared’, while adjacent ones have turned to fibrofatty connective tissue? Why is the heart relatively spared until quite late in the disease process? DMD therapeutics may require multidrug regimens, yet such multidrug approaches pose challenges with regard to both pharmaceutical development and regulatory pathways.

## Conflict of interest

EPH is cofounder and stockholder in ReveraGen BioPharma, cofounder and stockholder in AGADA BioSciences, and cofounder and stockholder in TRiNDS LLC.
